# Lateral Positioning and Airway Management in Penetrating Abdominal Trauma: A Case Report

**DOI:** 10.7759/cureus.78466

**Published:** 2025-02-03

**Authors:** Sanjay K Meena, Sharmishtha Pathak, Abhishek Singh, Nisha Jain

**Affiliations:** 1 Anaesthesiology, All India Institute of Medical Sciences, New Delhi, IND

**Keywords:** airway management, difficult airway management, lateral position, penetrating thoracoabdominal trauma, video laryngoscopy (vl)

## Abstract

Airway management in trauma patients is a critical component of resuscitation, often complicated by the unique positioning required by the nature and location of injuries. Penetrating abdominal trauma requiring lateral positioning presents an uncommon and challenging scenario for airway stabilization, particularly when traditional supine approaches are contraindicated.

We describe the case of an 18-year-old male patient who was taken to the operating theater with a penetrating abdominal injury necessitating lateral positioning due to ongoing bleeding and hemodynamic instability. Initial airway assessment revealed a compromised airway, hypoxia, and a risk of aspiration, necessitating rapid airway intervention in the lateral position. Using a video laryngoscope, a secure airway was successfully established while maintaining the patient's position. The lateral approach enabled optimal management of both the penetrating injury and airway compromise, preventing further exacerbation of the injury and reducing aspiration risks.

This case highlights the challenges and considerations in managing airways in trauma patients who cannot tolerate supine positioning. It underscores the importance of adaptability in emergency airway techniques, the utility of advanced tools for visualization and intubation, and the value of multidisciplinary collaboration in managing complex trauma scenarios.

## Introduction

Penetrating abdominal trauma is a critical injury that poses immediate threats to life, including hemorrhage, peritonitis, and organ damage. Effective management requires rapid assessment and intervention to stabilize the patient while addressing life-threatening injuries. Airway management is a cornerstone of resuscitation in trauma, as ensuring adequate oxygenation and ventilation is paramount to preventing hypoxia and secondary complications [1].

Traditional airway management techniques are typically performed with the patient in the supine position to facilitate optimal access and visualization of the airway. However, certain trauma scenarios, such as those involving complex injuries or anatomical considerations, may necessitate alternative patient positioning [2]. The lateral position, while less commonly employed in trauma settings, may be required to manage specific injury patterns, control hemorrhage, or prevent exacerbation of existing injuries.

Managing the airway in the lateral position introduces unique challenges, including altered anatomical orientation, reduced airway visualization, and increased difficulty in securing endotracheal intubation. Advanced airway tools and techniques, such as video laryngoscopy (VL) or fiberoptic bronchoscopy (FOB), may be required to overcome these obstacles. Studies have demonstrated that VL improves visualization and first-pass success rates, making it a valuable tool in challenging airway scenarios [3,4]. Despite these complexities, literature on airway management in non-supine trauma patients is sparse, particularly in cases involving penetrating abdominal injuries.

This case report describes the successful airway management of a patient with penetrating abdominal trauma who required lateral positioning. The report aims to highlight the challenges, considerations, and techniques employed to achieve a secure airway in this unconventional scenario, providing insights for clinicians facing similar situations.

## Case presentation

**Figure 1:**
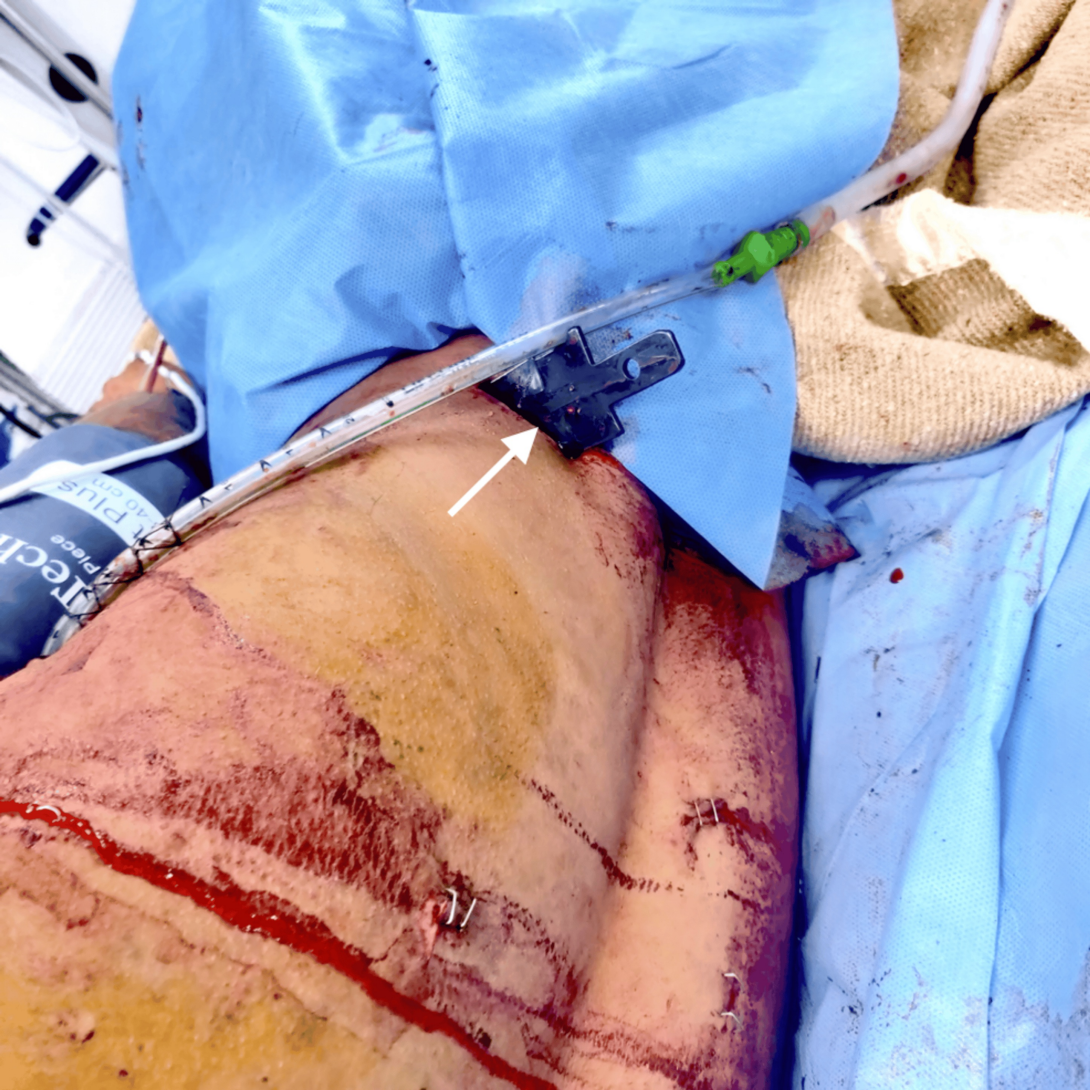
Impaled metallic knife (white arrow) in the dorsal aspect of the right lower quadrant of the abdomen

An 18-year-old male patient, weighing 70 kg and measuring 170 cm in height, presented to the emergency department with penetrating trauma to the chest and abdomen, accompanied by an in situ metallic knife located on the dorsal aspect of the right lower abdominal quadrant (Figure 1).

**Figure 2:**
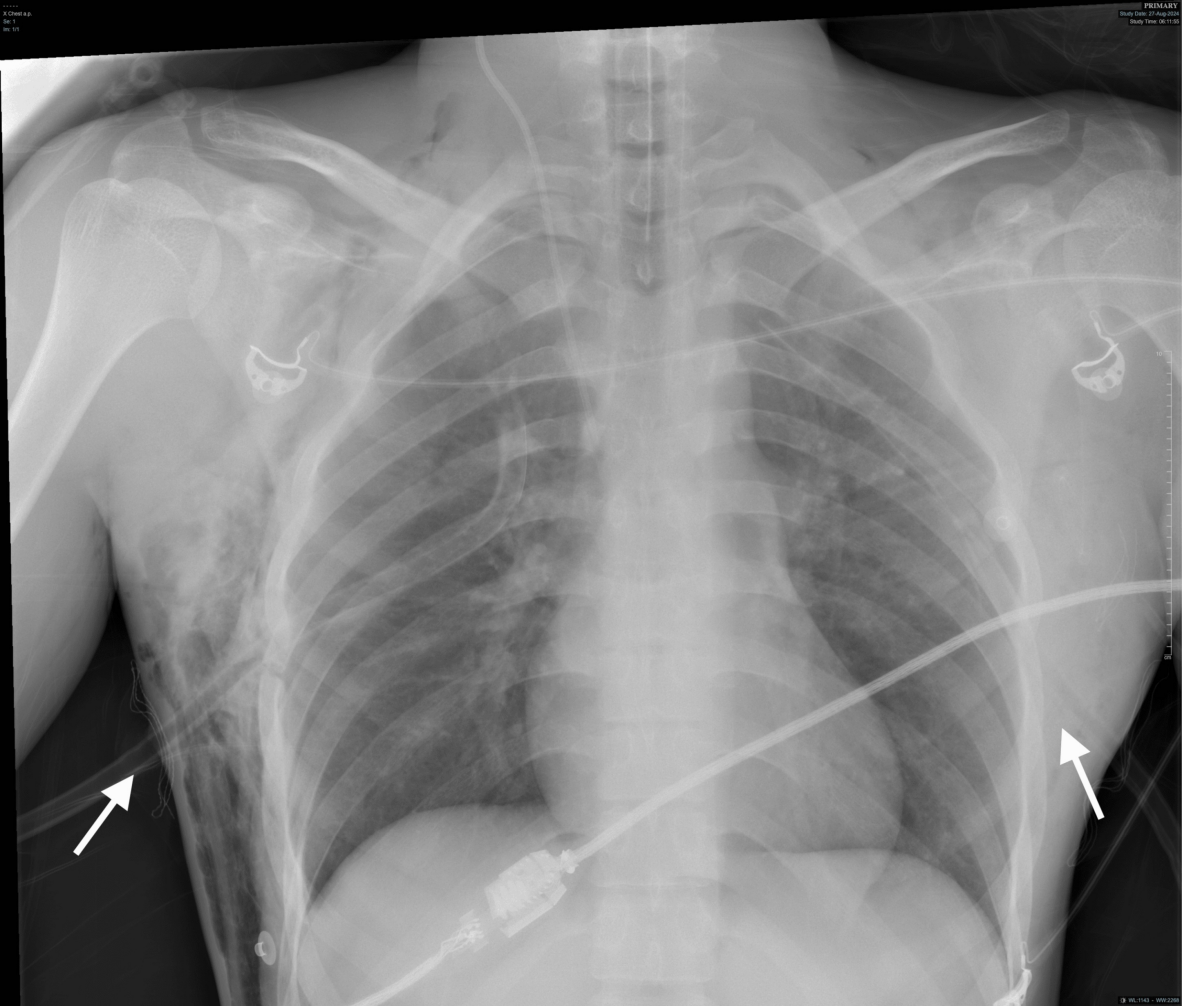
Chest X-ray showing the presence of bilateral intercostal drainage tubes (white arrows)

The primary survey revealed bilateral pneumothorax, confirmed by PneumoScan. Bilateral intercostal drains (ICDs) were inserted, with a gush of air noted on the right side (Figure 2).

**Figure 3:**
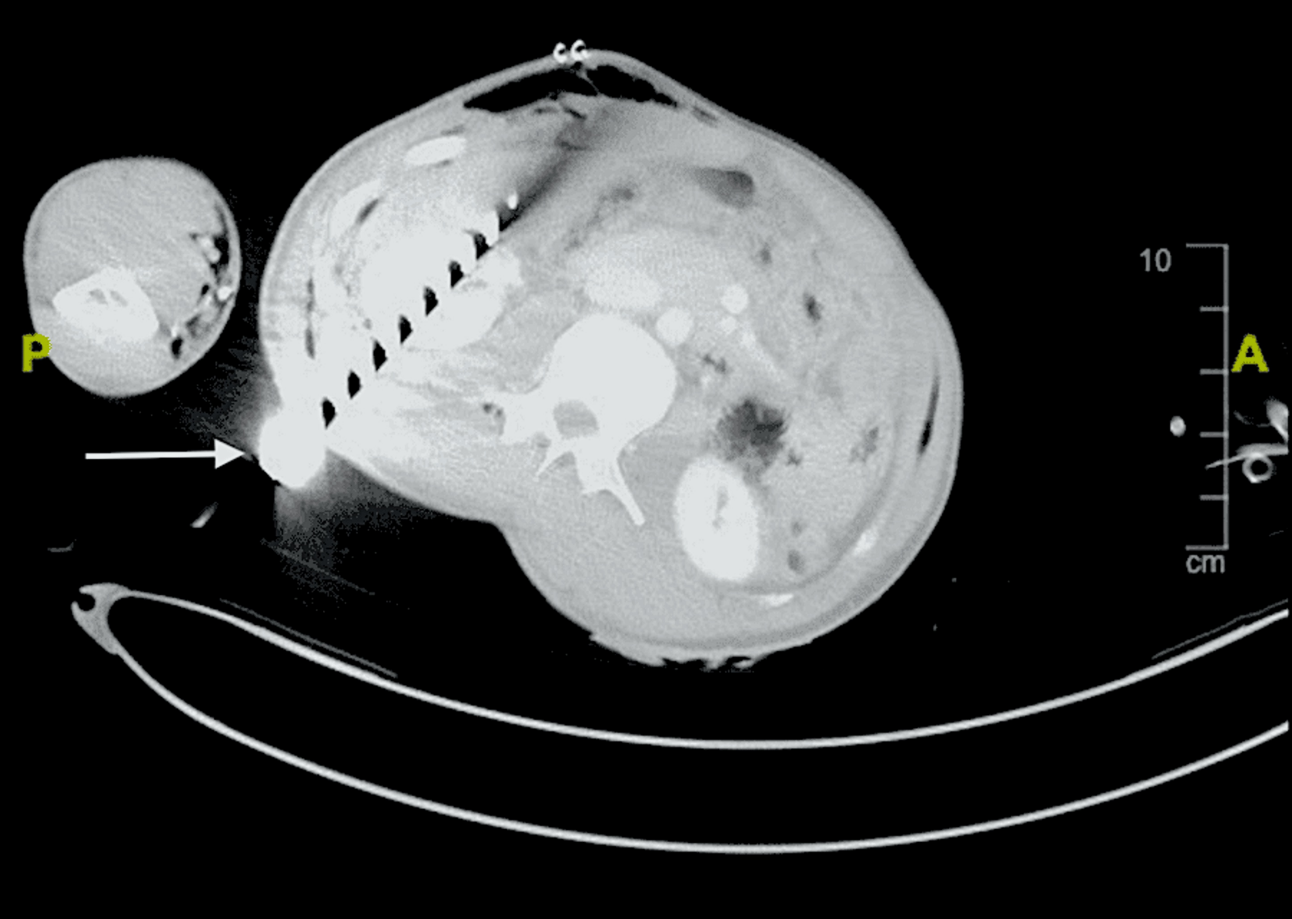
CT scan of the abdomen showing impaled sharp knife (white arrow) through the lower pole of the right kidney sparing the right renal pedicle CT: computed tomography

The patient had multiple stab wounds of varying sizes across the body, with a metallic knife impaled in the right lower quadrant of the abdomen. The CT scan revealed a metallic knife impaled at the lower pole of the right kidney, causing a renal laceration measuring 5 × 2 cm and a liver laceration of 3 × 1 cm (Figure 3).

**Figure 4:**
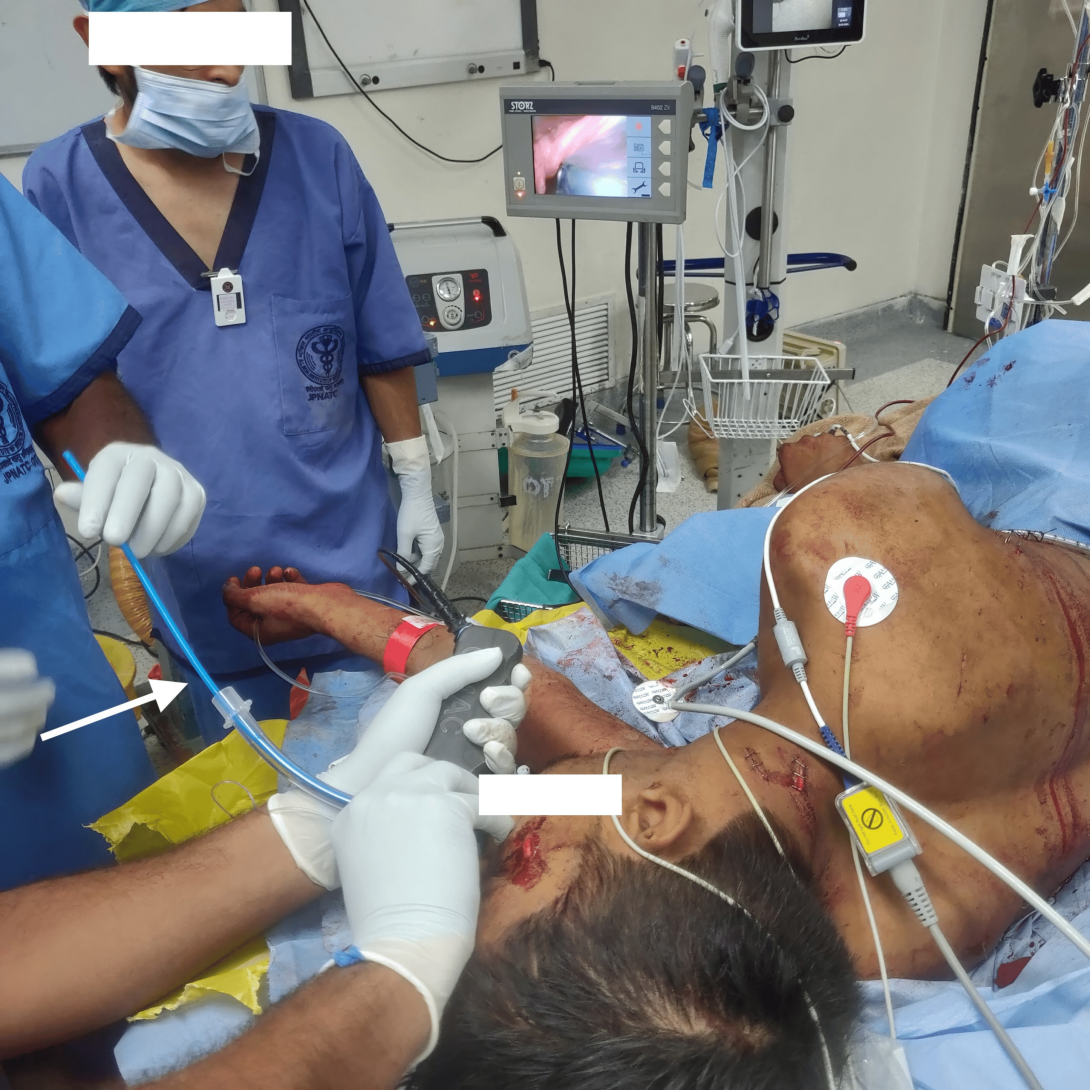
Bougie-guided (white arrow) intubation performed with the help of a C-MAC® video laryngoscope

Following a multidisciplinary evaluation, it was determined that the surgical removal of the knife would proceed under general anesthesia. Given the life-threatening risks of knife dislodgement, the traditional supine position was deemed inappropriate for airway management. Instead, a customized approach was adopted, prioritizing the use of a C-MAC® video laryngoscope (Karl Storz SE & Co. KG, Tuttlingen, Germany) in the lateral position to secure the airway. A supraglottic airway device was prepared as a backup, and a surgical airway was reserved as the last resort. The patient was transferred to the operating room, where monitoring, including ECG, non-invasive blood pressure, and pulse oximetry, was initiated. The initial airway examination revealed adequate mouth opening, neck mobility, a Mallampati score of II, and the absence of anatomical abnormalities that could complicate intubation. Preoxygenation was carried out with 100% oxygen for three minutes. Modified rapid sequence induction was performed using ketamine and rocuronium. Airway management in the left lateral position was successfully achieved using the C-MAC® video laryngoscope with bougie guidance, which revealed a Cormack-Lehane grade 2b glottic view, facilitating the insertion of an 8 mm cuffed orotracheal tube (Figure 4).

Throughout the procedure, the knife remained undisturbed, ensuring patient safety. The surgical procedure involved the careful removal of the metallic knife, repair of the liver and right kidney lacerations, and closure of the abdominal muscles with hemostasis. The patient remained hemodynamically stable throughout the surgery with minimal blood loss and adequate urine output. Postoperatively, the patient was extubated and shifted to the recovery room with supplemental oxygen via a face mask. On postoperative day 4, bilateral ICDs were removed after ensuring no residual collection. The patient was discharged on postoperative day 7 with stable vital signs. At a one-month follow-up, the patient was doing well and exhibited no residual complications.

## Discussion

Penetrating trauma to the chest and abdomen presents significant challenges in both anesthesia and surgical management, requiring a multidisciplinary approach [5]. This case highlights the complexities of managing an impaled object in a critical anatomical region and the necessity for meticulous airway management, stabilization, and surgical intervention. The patient's presentation in a lateral position due to the impaled knife was unique and required specific adaptations in airway management and surgical techniques. Airway management is particularly challenging when the presence of a projecting foreign body precludes supine positioning, as any inadvertent manipulation of the object can precipitate hemodynamic collapse. Such cases demand meticulous planning with advanced airway management techniques.

Airway management in penetrating trauma cases is critical for ensuring oxygenation and preventing secondary complications. In this case, the decision to intubate the patient in the lateral position was necessitated by the location and orientation of the impaled knife. Intubation in unconventional positions can be challenging, but the use of a C-MAC® video laryngoscope and bougie facilitated successful intubation on the first attempt. Studies have shown that VL improves visualization and increases the likelihood of first-pass success, particularly in cases involving anatomical distortion or restricted airway access [4]. Lateral-positioned endotracheal intubation proved to be a safe and effective technique in this life-threatening situation involving a projecting foreign object in the abdomen. While FOB can assist in identifying the tracheal lumen and enable smoother endotracheal tube placement, it may not be feasible in emergency situations or when the patient is in a lateral position. Additionally, blood and oral secretions can obstruct visualization, limiting its utility. Laryngeal mask airways are less invasive, quicker to deploy, and suitable for short-term airway management in the lateral position but may be less effective in maintaining a secure airway for high-risk patients. Surgical airways are definitive and bypass upper airway challenges but require advanced skills, carry a higher risk of complications, and are more difficult to perform in the lateral position.

The use of a modified rapid sequence induction with ketamine and rocuronium was appropriate, given the patient's hemodynamic status and potential for airway compromise. Ketamine is well-documented for providing both analgesia and hemodynamic stability, making it a suitable agent in trauma cases, while rocuronium ensures adequate muscle relaxation for intubation and surgical intervention [6].

The surgical approach to penetrating trauma must be carefully planned to minimize further injury and ensure hemostasis. In this case, the impaled knife caused significant renal and liver lacerations, as well as pneumoperitoneum, necessitating precise removal and repair. The removal of impaled objects should always be performed in a controlled surgical setting to prevent exacerbation of bleeding or additional damage to surrounding tissues. Timely repair of organ lacerations, along with meticulous closure of the abdominal wall, was essential in achieving a favorable outcome for this patient [7]. Bilateral pneumothorax, a common complication in penetrating chest trauma, was effectively managed with bilateral intercostal drains. The timely insertion of these drains prevented further compromise of respiratory function [8].

The successful outcome in this case can be attributed to early stabilization, judicious airway management, effective surgical intervention, and comprehensive postoperative care. This report adds to the growing body of evidence on managing penetrating trauma in unconventional scenarios, emphasizing the importance of adaptability, advanced equipment, and a systematic approach to care.

## Conclusions

This case underscores the importance of a systematic and multidisciplinary approach to managing penetrating trauma involving critical anatomical structures. The successful management of an impaled knife in the abdomen, along with associated complications such as pneumothorax and organ lacerations, required a combination of advanced airway techniques, surgical precision, and comprehensive postoperative care. The use of VL and a bougie for intubation in the lateral position highlights the importance of adaptability and specialized equipment in managing difficult airways. Early stabilization, timely surgical intervention, and close postoperative monitoring were crucial factors in ensuring a favorable outcome. This report emphasizes the need for tailored strategies in unique trauma scenarios and contributes to the growing body of knowledge on the management of impalement injuries.
